# Endophytic fungal communities of *Calicotome spinosa*—an important medicinal plant of Tizi-Ouzou (Algeria)

**DOI:** 10.1007/s13353-025-00980-6

**Published:** 2025-06-03

**Authors:** Amina Zareb, Piotr Banachewicz, Polina Havrysh, Lidia Błaszczyk, Tinhinane Hammad, Cylia Meftah, Sylwia Salamon

**Affiliations:** 1 Department of Ecology and Environment. Faculty of Biological Sciences and Agronomical Sciences, Mouloud Mammeri University, Tizi-Ouzou, Algeria; 2 Department of Plant Microbiomics, Institute of Plant Genetics, Polish Academy of Sciences, Poznań, Poland

**Keywords:** *Calicotome spinosa*, Diversity, Endophytic fungi, Phyllosphere, Tizi-Ouzou

## Abstract

Endophytic fungi enhance plant growth and improve tolerance to abiotic and biotic stress. *Calicotome spinosa* is an important medicinal plant that occupies the area of Tizi-Ouzou and is considered a nonflammable plant that maintains this ecosystem and regenerates degraded substrates. However, the knowledge about the endogenous fungal assembly of *C. spinosa* remains underexplored. Therefore, this study aimed to isolate and determine the fungal communities inhabiting the *C. spinosa* leaves endosphere using morphological and molecular identification methods. Morphological identification revealed the presence of *Alternaria*, *Cladosporium*, *Mucor*, *Neoscytalidium*, *Rhizoctonia*, *Rhodotorula*, *Phoma*, *Penicillium*, and *Trichophyton* genera. Molecular analysis of the selected phylogenetic markers’ nucleotide sequences detailed the previous one and discovered the following: *Biscogniauxia mediterranea*, *Schizophyllum commune*, *Penicillium polonicum*, *Athelia bombacina*, *Chaetomium globosum*, *Penicillium brevicompactum*, *Coriolopsis* sp., *Coprinellus* sp., *Aspergillus chevalieri*, *Aspergillus wentii, Canariomyces microspores*, *Alternaria alternata*, *Canariomyces notabilis*, *Rosellinia* sp., *Penicillium* sp., and *Alternaria* sp. The taxonomic knowledge gained here for the first time about the group of endophytic fungi associated with the ethnomedicinal plant growing in Algeria, and the disposition of these fungi isolates provides a basis for further research into understanding the functions they may have in the plant. These isolates are therefore a source of potential candidates for further research into their possible applications as beneficial microorganisms in ecology, environmental and plant protection, and sustainable agriculture.

**Supplementary Information:**

The online version contains supplementary material available at 10.1007/s13353-025-00980-6.

## Introduction

Tizi-Ouzou is a mountainous region where summer temperatures can reach 50 °C, resulting in recurrent fires during July–August. *Calicotome spinosa*, a resilient plant, thrives in these harsh environments and is known for its ability to maintain the ecosystem and regenerate degraded soils. This heliophilous and xerophilic plant is found in a variety of habitats, ranging from coastal areas to inland regions, all of which are marked by hot and arid climates (Bonis 2008). Moreover, due to its flavonoid content, this medicinal plant possesses antioxidant and anti-inflammatory properties (Larit et al. 2012).

Endophytic fungi, which inhabit plant tissues without causing disease, establish symbiotic relationships with their hosts (Bavya et al., 2021). These fungi help plants adapt to environmental stressors, such as temperature extremes, pH fluctuations, salinity, and heavy metal contamination, while also producing bioactive metabolites that protect against pests and pathogens (Pereira et al. 2022).

The preliminary study presented here aims to catalogue the culturable endophytic fungi associated with *Calicotome spinosa*. Despite their crucial role in plant health and stress tolerance, endophytes in North African medicinal plants remain underexplored. By investigating the endophytic community of *C. spinosa*, it is expected that new fungal strains will be identified that can enhance the plant’s medicinal potential or serve as sources of bioactive compounds. The acquisition and appropriate taxonomic classification of these endophytes could lead to new biotechnological applications in medicine, agriculture, and environmental sustainability.

## Materials and methods

### Plant material sampling and preparation

Leaves were collected from six healthy *Calicotome spinosa* plants growing in the natural vegetation of the Ouaguenoun region (Tizi-Ouzou, Algeria; Supplementary Fig. 1). Thirty fresh and healthy leaves were randomly harvested and pooled for each plant, resulting in 180 leaves in total.

Surface sterilization was performed according to the protocol described by Helander et al. (1994). Following sterilization, three leaves were placed on each Petri dish containing Potato Dextrose Agar (PDA) supplemented with amoxicillin (1000 mg; GlaxoSmithKline, Algeria) to inhibit bacterial growth. The plates were incubated at 23–27 °C for up to two months. Emerging fungal colonies were successively subcultured on fresh PDA to obtain pure isolates. These isolates were subsequently used for morphological and molecular identification, as well as for preservation for future studies. Strains identified based on morphology were subjected to thermal stress up to 50 °C for one month.

### Morphological and molecular identification

Fungal isolates were initially characterized based on their macroscopic and microscopic features. From the 60 strains identified based on morphology, 16 that exhibited thermotolerance (survival at up to 50 °C) were chosen for further molecular analysis. The selection was guided by existing knowledge of their potential ecological roles, particularly in providing protection against abiotic and biotic stresses.

Pure cultures were maintained on PDA at 24 °C. Genomic DNA was extracted from 15-day-old mycelia using the Wizard® Genomic DNA Purification Kit (Promega, USA). Extracted DNA was used for PCR amplification of phylogenetic markers, including the internal transcribed spacer (ITS) region, beta-tubulin (*tub2/BenA*), translation elongation factor 1-alpha (*tef1*), small subunit (SSU), and large subunit (LSU) rDNA. Amplification and sequencing were conducted using the Sanger method, following the protocol described by Salamon et al. (2020). The sequences of the primers and PCR conditions are listed in Supplementary Table 1. Raw sequence data were analyzed with Chromas Lite software (version 2.6), and fungal identification was carried out via BLAST searches against the GenBank nucleotide database (NCBI). The obtained sequences were deposited in the NCBI GenBank database, and their accession numbers are provided in Table 1.

**Table 1:** Morphological and molecular endogenous fungi identified from the *C. spinosa* leaves from the region of Ouaguenoun wilaya of Tizi-Ouzou identified with the use of a molecular approach

Morphological identification				
Genus	Phylum	Class	Order	
*Alternaria*	*Ascomycota*	^Dothideomycetes^	Pleosporales	
***Cladosporium***	*Ascomycota*	^Dothideomycetes^	Capnodiales	
*Mucor*	*Mucoromycota*	Mucoromycetes	Mucorales	
*Neoscytalidium*	*Ascomycota*	^Dothideomycetes^	Botryosphaeriales	
*Penicillium*	*Ascomycota*	^Eurotiomycetes^	Eurotiales	
*Phoma*	*Ascomycota*	^Dothideomycetes^	Pleosporales	
***Rhizoctonia***	*Basidiomycota*	^Agaricomycetes^	Cantharellales	
*Rhodotorula*	*Basidiomycota*	Microbotryomycetes	Sporidiobolales	
***Trichophyton***	*Ascomycota*	^Eurotiomycetes^	Onygenales	
*NIS*	/			
Molecular identification				
Species	Phylum	Class	Order	GenBank ID
*Alternaria alternate*	*Ascomycota*	Dothideomycetes	Pleosporales	PP510403.1
				PP510424.1
				PP510414.1
*Alternaria* sp.	*Ascomycota*	Dothideomycetes	Pleosporales	PP510392.1
				-
				PP510408.1
*Aspergillus chevalieri*	*Ascomycota*	Eurothiomycetes	Eurotiales	PP510399.1 PP510400.1
				PP510422.1
				-
*Aspergillus wentii*	*Ascomycota*	Eurothiomycetes	Eurotiales	PP510401.1
				PP510423.1
				PP510412.1
*Athelia bombacina*	*Basidiomycota*	Agaricomycetes	Atheliales	PP510394.1
				PP510418.1
				-
*Biscogniauxia mediterranea*	*Ascomycota*	Sordariomycetes	Xylariales	PP510390.1
				PP510416.1
				PP510407.1
*Canariomyces microsporus*	*Ascomycota*	Sordariomycetes	Sordariales	PP510402.1
				-
				PP510413.1
*Canariomyces notabilis*	*Ascomycota*	Sordariomycetes	Sordariales	PP510404.1
				PP510425.1
				PP510415.1
*Chaetomium globosum*	*Ascomycota*	Sordariomycetes	Sordariales	PP510395.1
				PP510419.1
				PP510409.1
*Coprinellus* sp.	*Basidiomycota*	Agaricomycetes	Agaricales	PP510398.1
				PP510421.1
				PP510411.1
*Coriolopsis* sp.	*Basidiomycota*	Agaricomycetes	Polyporales	PP510397.1
				-
				PP510410.1
*Penicillium brevicompactum*	*Ascomycota*	Eurothiomycetes	Eurotiales	PP510396.1
				PP510420.1
				-
*Penicillium polonicum*	*Ascomycota*	Eurothiomycetes	Eurotiales	PP510393.1
				-
				-
*Penicillium* sp.	*Ascomycota*	Eurothiomycetes	Eurotiales	PP510406.1
				-
				-
*Rosellinia* sp.	*Ascomycota*	Sordariomycetes		PP510405.1
				PP510426.1
				-
*Schizophyllum commune*	*Basidiomycota*	Agaricomycetes	Agaricales	PP510391.1
				PP510417.1
				-

The most dominant genera are in bold

## Results

### Morphological identification of endophytic fungi

In this study, a total of 60 fungal isolates were obtained from the endosphere of leaf tissues of six healthy *Calicotome spinosa* plants and taxonomically assigned based on combined morphological and molecular analyses. The isolates belonged to three major fungal phyla: Ascomycota, Basidiomycota, and Mucoromycota.

**Fig. 1:**
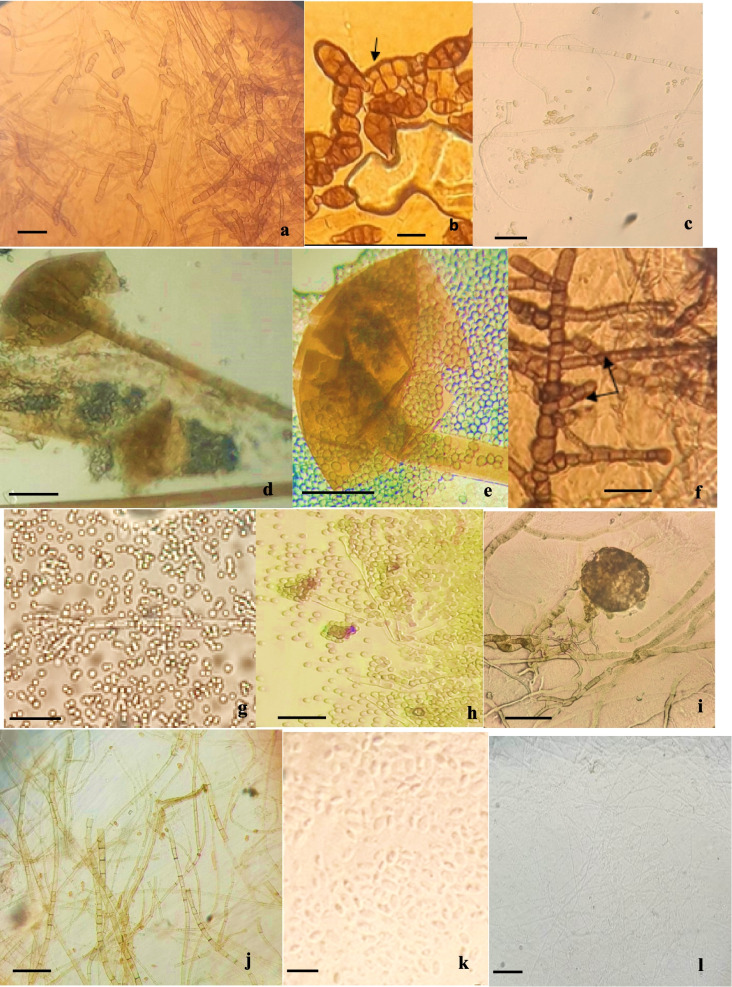
Microscopic morphology of endophytes isolated from *C. spinosa* under magnification (Gr × 400): **a** and **b** – conidia (**a**) and hyphae (**b**) of *Alternaria;* the arrow indicates the hyphae; **c** – hyphae and spores of *Cladosporium*. 5 µm scale bars: **d** and **e**—sporangium and sporangiospores of *Mucor*; **f**—hyphae of *Neoscytalidium;* arrows indicate hyphae. 10 µm scale bars: **g** and **h**—conidiophore and spores of *Penicillium*; **i**—hypha and pycnidium of *Phoma*; **j**—hyphae of *Rhizoctonia*; **k**—blastospores of *Rhodotorula*; **l** – hyphae of *Trichophyton*

Within Ascomycota, the endophytes were assigned to seven distinct genera, encompassing 18 orders and six classes, including Dothideomycetes, Eurotiomycetes, and Sordariomycetes. For Basidiomycota, the isolates were classified into two classes: Agaricomycetes and Microbotryomycetes. A single class, Mucoromycetes, was represented within Mucoromycota (Fig. 1). Additionally, several isolates were grouped as nonidentified strains (NIS).

### Molecular identification of endophytic fungi

Of the 60 isolates, 16 thermotolerant strains were selected for in-depth molecular identification. Among these, 11 strains were identified at the species level and five at the genus level. Most of these microorganisms belonged to Ascomycota, supporting the earlier microscopic identification results.

Only four isolates were classified within Basidiomycota, including *Athelia bombacina*, *Coprinellus* sp., *Coriolopsis* sp., and *Schizophyllum commune*. Notably, 12 isolates that could not be reliably classified by morphological characteristics alone were successfully identified through molecular methods. These included *Aspergillus chevalieri*, *Aspergillus wentii*, *Athelia bombacina*, *Biscogniauxia mediterranea*, *Canariomyces microsporus*, *Canariomyces notabilis*, *Chaetomium globosum*, *Coprinellus* sp., *Coriolopsis* sp., *Rosellinia* sp., and *Schizophyllum commune.*

Table 1 and Supplementary Fig. 2 comprehensively overview all endophytic fungal strains isolated from *C. spinosa* identified in this study.

## Discussion

This study provides the first report on the diversity of endophytic fungi associated with *Calicotome spinosa*, offering valuable baseline data for future microbial ecology research on North African medicinal flora. The results reveal a diverse assemblage of culturable endophytic fungi in *C. spinosa* leaves, predominantly representing taxa from the phylum Ascomycota. This finding is consistent with previous studies, which indicate that Ascomycota comprise approximately 73.1% of known endophytic fungal taxa (Materatski et al. 2018). Members of Ascomycota have been shown to possess a greater number of genes linked to nutrient acquisition, carbohydrate metabolism, competitive ability, and resistance to both abiotic and biotic stressors, compared to other fungal groups (Dos Reis et al. 2023). In addition, many Ascomycota species act as soil saprophytes, capable of degrading complex organic matter and thus playing a central role in ecosystem-level nutrient cycling (Zuo et al. 2022).

Previous studies have documented numerous endophytic fungi associated with Fabaceae plants, including various genera such as *Alternaria*, *Aspergillus*, *Fusarium*, *Penicillium*, *Xylaria*, *Epicoccum*, and *Phomopsis*, which produce a variety of secondary metabolites (Saini et al. 2025). Consistent with these findings, our study identified several fungal genera reported in Fabaceae endophyte communities, such as *Aspergillus*, *Cladosporium*, *Chaetomium*, *Penicillium*, and *Rosellinia*. This overlap highlights that *C. spinosa*, belonging to the Fabaceae family, might host fungal symbionts with biotechnological applications. The discovery of both widely recognized metabolite-producing taxa and lesser-known or underexplored genera emphasizes the ecological and functional diversity of its endophytic community.

Several fungal genera identified as endophytes in *C. spinosa* leaves are known for diverse ecological roles, ranging from saprophytism to latent pathogenicity. However, almost none of the fungi detected in the endosphere of *C. spinosa* are known to be pathogenic to legumes (Sbai et al. 2024); instead, they are recognized as pathogens of other species. Dermatophyte pathogen *Trichophyton* was found ubiquitously in leaf tissues, although it is rarely reported as a plant endophyte (Kaufman et al. 2007). A notable example of a latent pathogen in our study is *Rhizoctonia*, the second most abundant genus in *C. spinosa* leaves. Despite its presence, no disease symptoms were observed, indicating a potential endophytic or latent interaction. *Rhizoctonia solani* is known to infect over 27 plant families, including important Fabaceae species such as soybeans, peanuts, beans, and alfalfa (Nasimi et al. 2024), while *R. medicaginis* specifically affects *Medicago sativa* (Ogoshi 1987). Similarly, *Alternaria alternata*, the most frequently isolated species in this study, is a common endophyte and occasional pathogen in various plants, including halophytes (Zuo et al. 2022). Its dominance in *C. spinosa* may be attributed to its adaptability and ecological versatility. Other genera detected, such as *Rosellinia* and *Canariomyces*, are also known for their pathogenic potential. Their presence in asymptomatic leaves supports the idea that certain pathogenic fungi may persist as endophytes under nonstressful conditions, possibly gaining a survival advantage in arid or fluctuating environments. Such associations could provide adaptive benefits like thermotolerance and drought resistance, contributing to host survival and ecosystem resilience (Zuo et al. 2022). *Penicillium brevicompactum*, a xerophilic fungus often associated with indoor environments, and *Aspergillus* species, which are capable of growth under low water availability (Ndagijimana et al. 2008; Bekoe et al. 2021), further highlight the resilience of the endophytic community in *C. spinosa* to drought-prone Mediterranean habitats. *Schizophyllum commune*, typically found on softwood and silage (Tovar-Herrera et al. 2018), was also detected, but in the absence of disease symptoms, it is likely functioning here as a non-pathogenic endophyte.

## Conclusion

This study presents the first report on the diversity of endophytic fungi associated with the medicinal plant *Calicotome spinosa*. The dominant genera identified in this study *Trichophyton*, *Rhizoctonia*, *Alternaria*, and *Cladosporium* belong predominantly to the phylum Ascomycota, which also represented the most taxonomically diverse group across all isolates. Interestingly, the presence of Basidiomycota and Mucoromycota, although limited, expands the known taxonomic breadth of endophytes in Fabaceae plants. The use of both morphological and molecular methods allowed for the accurate identification of 16 thermotolerant strains, some of which (e.g., *Athelia bombacina* and *Schizophyllum commune*) are rarely reported as endophytes. These findings suggest that *C. spinosa* may serve as a reservoir for diverse and potentially stress-tolerant fungal symbionts. The prevalence of genera known for their ecological plasticity and functional versatility may contribute to the plant’s resilience in Mediterranean environments. Overall, the high diversity of endophytic fungi identified in this study highlights their ecological importance and biotechnological potential, pointing to promising avenues for future functional and applied research.

## Supplementary Information

Below is the link to the electronic supplementary material.

Supplementary file1 (DOCX 2030 KB)

## Data Availability

The DNA sequences obtained in this study have been deposited in GenBank and are available under the following accession numbers: PP510403.1, PP510424.1, PP510414.1, PP510392.1, PP510408.1, PP510399.1, PP510400.1, PP510422.1, PP510401.1, PP510423.1, PP510412.1, PP510394.1, PP510418.1, PP510390.1, PP510416.1, PP510407.1, PP510402.1, PP510413.1, PP510404.1, PP510425.1, PP510415.1, PP510395.1, PP510419.1, PP510409.1, PP510398.1, PP510421.1, PP510411.1, PP510397.1, PP510410.1, PP510396.1, PP510420.1, PP510393.1, PP510406.1, PP510405.1, PP510426.1, PP510391.1, PP510417.1.
