# Supplementary material for: Endophytic fungal communities of Calicotome spinosa—an important medicinal plant of Tizi-Ouzou (Algeria)
Source: J Appl Genet. 2025 Jun 3;66(3):763–9. doi: 10.1007/s13353-025-00980-6 (PMC12367884; doi:10.1007/s13353-025-00980-6)
Supplement: Supplementary file 1 — Supplementary file1 (DOCX 2030 KB) [file 13353_2025_980_MOESM1_ESM.docx]

*
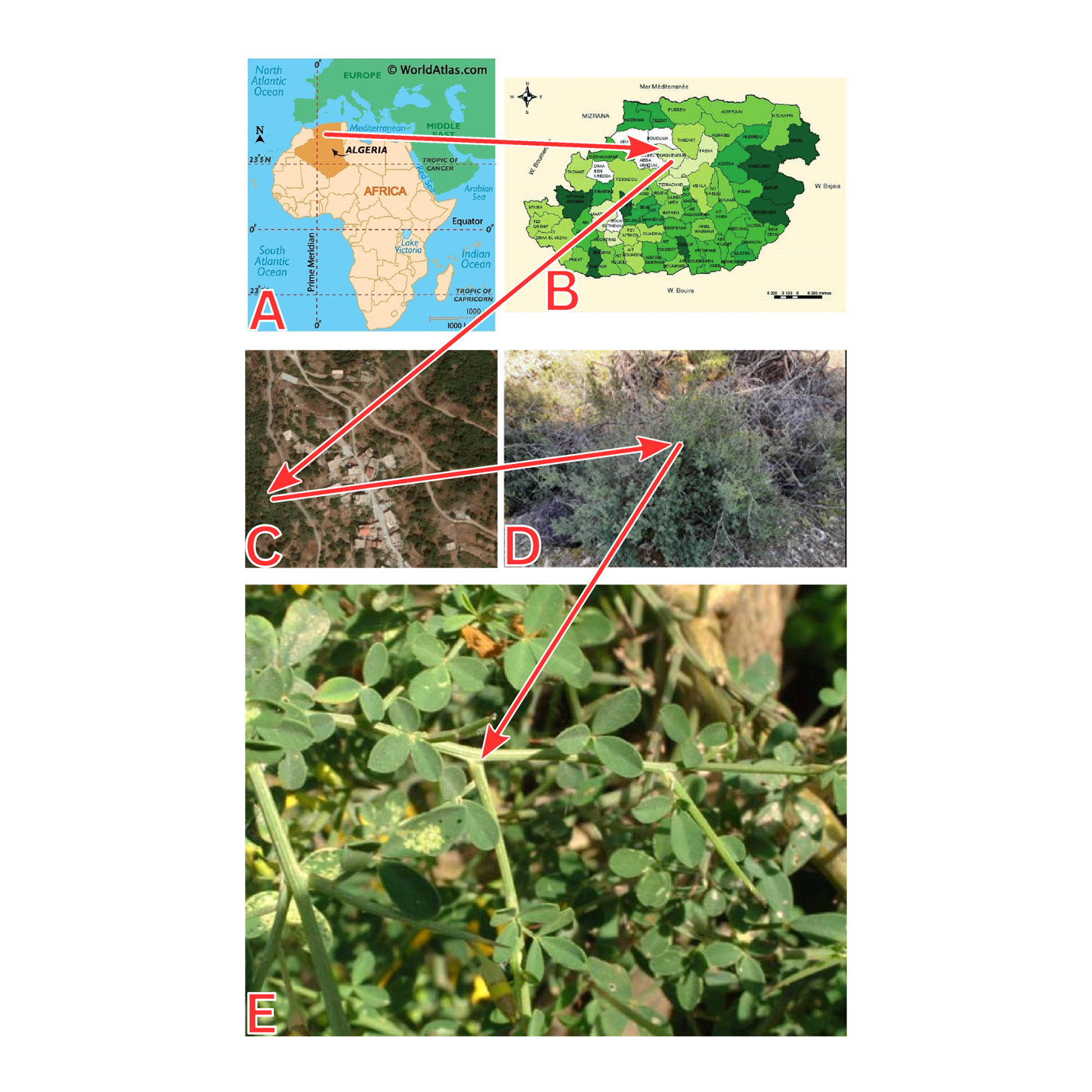
*

Supplementary Fig 1 The geographical location of the sampling site. (A-B) Ouaguenoun Tizi-Ouzou, Algeria. (C) View of the main site of the sampling (D-E) and details of *Calicotome spinosa.*


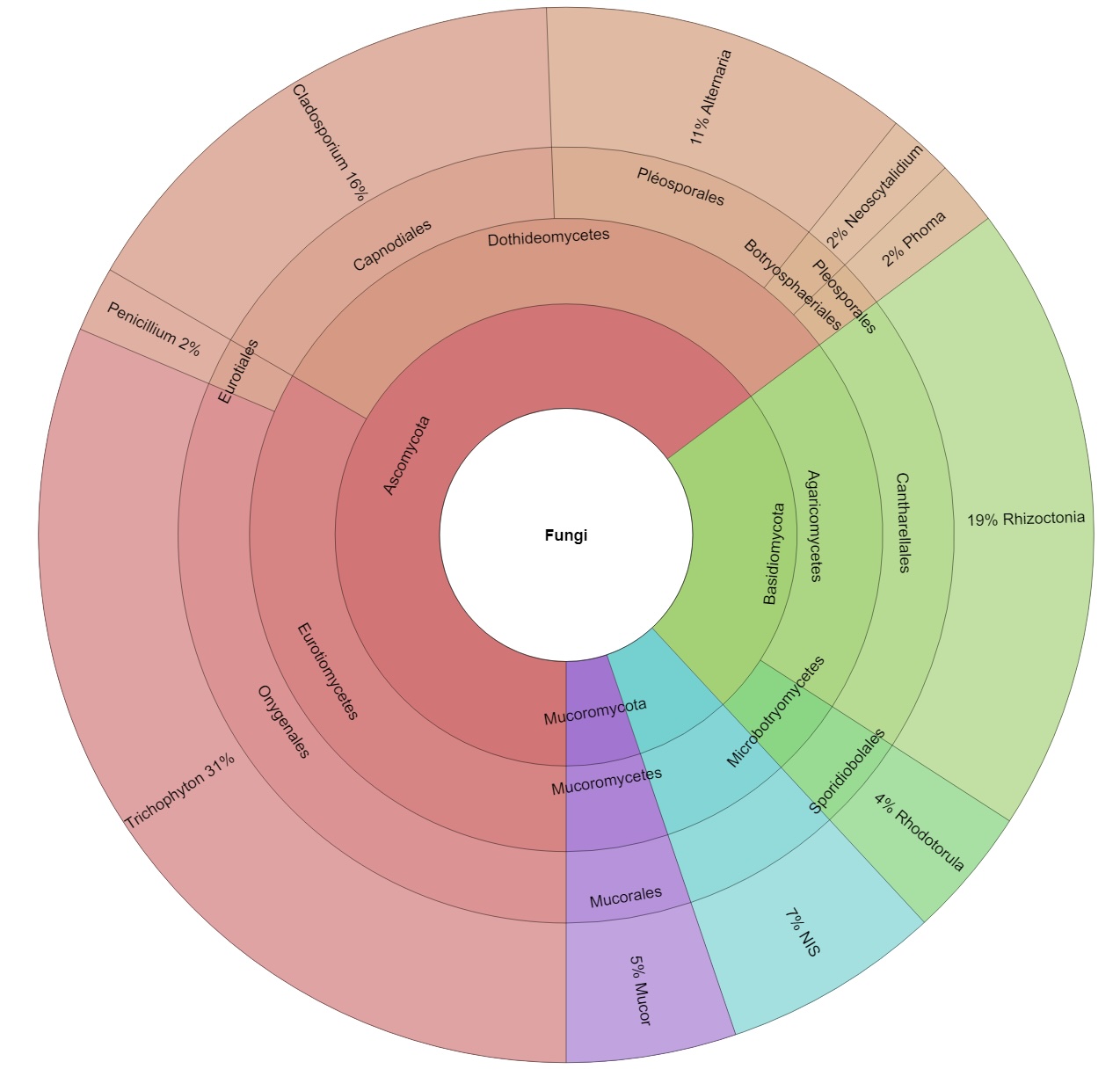


Supplementary Fig 2 The abundance of morphologically identified fungal endophytes in *C. spinosa* leaves

| Supplementary Table 1. Primers details and applied PCR conditions. | | | | |  |
| --- | --- | --- | --- | --- | --- |
| Locus | Primer description | Primer sequence 5'-3' | PCR conditions | Reference |  |
| Internal Transcribed Spacer (ITS) region of the rRNA | ITS1F | CTT GGT CAT TTA GAG GAA GTA A | 1. 95 ̊C – 5 min 2. 95 ̊C– 30 s 3. 52 ̊C– 30 s 4. 72 ̊C– 1 min 5. Steps 2–4 x 35 cycles 6. 72 ̊C– 8 min 7. 4 ̊C on hold | White et al. (1990) |  |
|  | ITS4 | TCC TCC GCT TAT TGA TAT GC |  |  |  |
| beta-tubulin (*tub2*/*BenA* | Bt2a | GGT AAC CAA ATC GGT GCT GCT TTC | 1. 95 ̊C – 5 min 2. 94 ̊C – 45 s 3. 55 ̊C – 45 s 4. 72 ̊C – 1 min 5. Steps 2-4 x 35 cycles 6. 72 ̊C – 7 min 7. 4 ̊C on hold | Glass and Donaldson (1995) |  |
|  | Bt2b | ACC CTC AGT GTA GTG ACC CTT GGC |  |  |  |
| Translation elongation factor 1-alfa (*tef1*) | ef1 | ATG GGT AAG GA(A/G) GAC AAG AC | 1. 95 ̊C – 8 min 2. 95 ̊C – 30 s 3. 53 ̊C – 60 s 4. 72 ̊C – 1 min 5. Steps 2-4 x 35 cycles 6. 72 ̊C – 5 min 7. 4 ̊C on hold | Geiser et al. (2004) |  |
|  | ef2 | GGA (G/A)GT ACC AGT (G/C)AT CATGTT |  |  |  |
| Small Subunit (SSU, 18S) of the rRNA | NS1 | GTA GTC ATA TGC TTG TCT C | 1. 95 ̊C – 5 min 2. 95 ̊C– 30 s 3. 52 ̊C– 30 s 4. 72 ̊C– 1 min 5. Steps 2–4 x 35 cycles 6. 72 ̊C– 8 min 7. 4 ̊C on hold | White et al. (1990) |  |
|  | NS4 | CTT CCG TCA ATT CCT TTA AG |  |  |  |
| Large Subunit (LSU, 28S) of the rRNA | LROR | ACC CGC TGA ACT TAA GC | 1. 95 ̊C – 5 min 2. 95 ̊C– 30 s 3. 52 ̊C– 30 s 4. 72 ̊C– 1 min 5. Steps 2–4 x 35 cycles 6. 72 ̊C– 8 min 7. 4 ̊C on hold | Vilgalys and Hester (1990)  Rehner and Samuels (1995) |  |
|  | LR6 | CGC CAG TTC TGC TTA CC |  |  |  |
